# Diversity and Vertical Distribution of *Planctomycetota* in the Water Column of the Remote North Pacific

**DOI:** 10.1111/1758-2229.70063

**Published:** 2025-02-20

**Authors:** Inês Rosado Vitorino, Nicola Gambardella, Miguel Semedo, Catarina Magalhães, Olga Maria Lage

**Affiliations:** ^1^ Department of Biology, Faculty of Sciences University of Porto Porto Portugal; ^2^ Interdisciplinary Centre of Marine and Environmental Research (CIIMAR/CIMAR) University of Porto Porto Portugal

**Keywords:** Illumina sequencing, PacBio sequencing, Pacific Ocean, Pelagic Environment, *Planctomycetota* diversity, ‘selfish’ behaviour

## Abstract

The extensive microbial diversity found in the oceans is becoming to be uncovered despite limited knowledge and cultured representatives for many taxonomic groups. This study analysed the distribution and diversity of *Planctomycetota* at four water column profiles of the Eastern North Pacific subtropical front (ENPSF) using 16S rRNA gene sequencing. A dual approach, utilising PacBio long‐reads and Illumina short‐reads, was employed to enhance the accuracy of taxonomic assignment and compare sequencing methods. The diversity of *Planctomycetota* increased below the deep chlorophyll maximum level (175–200 m) and in the mesopelagic layer (500 m), with beta‐diversity clustering distinctly separating samples according to different depths, resulting in pronounced vertical stratification. This community structure mirrors nutrient availability, as *P* *lanctomycetota* favour depths between 175 and 200 m, where high nitrate levels are present. More *P* *lanctomycetota* amplicon sequence variants (ASVs) were identified with PacBio than with Illumina, improving detection of these bacteria. Phylogenetic analyses performed after manual curation of ASVs led to the discovery of several unknown genera of *Planctomycetota*, indicating that substantial diversity within this group remains to be discovered and studied in remote oligotrophic oceans.

## Graphical abstract

In the remote regions of the Pacific Ocean, *Planctomycetota* diversity predominantly occupies deeper water layers ranging from 175 to 500 m. Phylogenetic analyses have identified an entire range of previously unknown genera within this group of bacteria.

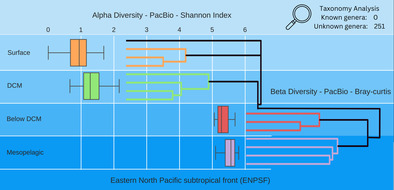

## 1 Introduction

The phylum *Planctomycetota* comprises a group of Gram‐negative bacteria with very peculiar characteristics, discovered approximately 100 years ago (Gimesi 1924). With the *Verrucomicrobiota* and *Chlamydiota* and other sister clades, they form the PVC superphylum (Wagner and Horn 2006). Their uniqueness is due to several features. These include (i) cell division through budding (Hirsch 1972) or binary fission (van Niftrik et al. 2009) lacking the bacterial common filamenting temperature‐sensitive mutant Z—FtsZ protein (Glockner et al. 2003), (ii) exhibiting a complex life cycle (Gade et al. 2005), (iii) possessing a complex cell plan characterised by extensive cytoplasmic membrane invagination (Lage, Bondoso, and Lobo‐da‐Cunha 2013), (iv) containing highly condensed DNA (Lage, Bondoso, and Lobo‐da‐Cunha 2013), (v) showing resistance to several classes of antibiotics (Cayrou, Raoult, and Drancourt 2010) and (vi) harbouring relatively large genomes with a high percentage of genes with unknown function (Rivas‐Marín and Devos 2018).

Members of *Planctomycetota* inhabit a ubiquitous range of ecosystems, colonising marine, freshwater, terrestrial, polluted and extreme environments (Lage et al. 2019). The diverse chemical, physical and biological characteristics of the mentioned environments have facilitated the development of varied metabolisms in these bacteria (Lage et al. 2019). Due to these features, *Planctomycetota* play important roles in the biogeochemical cycles. Primarily heterotrophs, they degrade organic matter of various types, namely many carbohydrates and even complex sulphated polysaccharides (Sun et al. 2021; Klimek, Herold, and Calusinska 2024). Their genomes can have an exceptionally high number of sulphatase genes (Glockner et al. 2003; Faria et al. 2018). They also further intervene in the nitrogen cycle through the anaerobic ammonium oxidation (anammox), the assimilation of ammonium and nitrate (van Niftrik and Jetten 2012) and nitrogen fixation in deep‐sea sediments (Kapili et al. 2020) and open surface ocean waters (Delmont et al. 2018). In marine environments, *Planctomycetota* have been described as part of marine snow, as they prefer a particle attached lifestyle (Salazar et al. 2015; Milici et al. 2017; Thompson, Valentine, and Peng 2024), in sediments (Lindh et al. 2017; Vitorino et al. 2022), and in deep ocean (Fuchsman et al. 2012; Storesund and Øvreås 2013). They were also found in association with several eukaryotes such as macroalgae (Bengtsson and Øvreås 2010; Bondoso et al. 2017), diatoms (Morris, Longnecker, and Giovannoni 2006; Bunse et al. 2016), sponges (Sheila et al. 2003; Sipkema et al. 2011; Izumi et al. 2013; Kallscheuer et al. 2020) and the giant tiger prawn (Fuerst et al. 1991). Furthermore, Zeigler Allen et al. (2012) detected a *Planctomycetota* bloom event in a marine upwelling site.

Since the development of the whole‐genome shotgun sequencing of microbial populations present in seawater (Venter et al. 2004), the microbial diversity inhabiting the open ocean is being elucidated in further global initiatives, including the Tara Ocean expeditions. Many new taxa, from phylum to species, have been discovered and how this vast community functions, interacts and affects our climate is beginning to be disclosed. Despite the widespread distribution of *Planctomycetota*, the ecological functions and interactions of this bacterial phylum in the open ocean remain poorly understood, emphasising the need for further research to elucidate their contributions to the ocean microbial communities and biogeochemical cycles.

In this present work, we investigated the water column vertical patterns of *Planctomycetota* and phylogenetic relationships in the Pacific remote oligotrophic ocean. The structure and diversity of the *Planctomycetota* community in one transect of the Eastern North Pacific subtropical front (ENPSF) was analysed through a dual‐sequencing approach, combining short‐read and long‐read amplicon sequencing for improved taxonomic assignment. The water samples covered the sunlit epipelagic waters (surface [5 m], deep chlorophyll maximum—DCM (108–130 m), below DCM [175–200 m]) and a part of the dark‐ocean (mesopelagic zone [500 m]), differing essentially in light availability, temperature, nitrate concentration and oxygen concentration.

## 2 Materials and Methods

### 2.1 Sampling Sites

Water column samples were collected in June 2018 in an oceanographic transect along the far edge of the ENPSF (Figure S1), 1000 nautical miles off the coast of Southern California, on board of the Schmidt Ocean Institute (SOI) research vessel ‘Falkor’, as previously described (Semedo et al. 2021). Briefly, a subset of 15 samples collected at different depths, far from each other from less than one degree of latitude, and within the upper 500 m of the water column, were used in this study (Table 1). A total of 3.75 L of seawater was filtered with a Sterivex filter (0.2 μm pore size) for microplankton analysis, stored on board at −80°C and transported in dry ice to CIIMAR laboratory for later DNA extraction. Samples were classified according to their depth and in situ chlorophyll concentrations. Four different depth layers were used in this study: surface (5 m, *n* = 4), deep chlorophyll maximum (DCM, 108–130 m, *n* = 4), below DCM (175–200 m, *n* = 3) and mesopelagic (500 m, *n* = 4). The DCM depths observed in this transact were similar to the DCM depths previously registered for the Pacific Ocean (Letelier et al. 2004; Sauzède et al. 2017).

**TABLE 1:** Sampling site coordinates and depth layers of samples used in this study.

Depth layer	Sample ID	Cast	Depth (m)	Latitude	Longitude
Surface (5 m)	S2_5m	5	5	30.757	−132.081
DCM (108–130 m)	S2_130m	5	130	30.757	−132.081
Mesopelagic (500 m)	S2_500m	5	500	30.757	−132.081
Surface (5 m)	S3_5m	4	5	30.255	−132.083
DCM (108–130 m)	S3_122m	4	122	30.255	−132.083
Below DCM (175–200 m)	S3_175m	4	175	30.255	−132.083
Mesopelagic (500 m)	S3_500m	4	500	30.255	−132.083
Surface (5 m)	S4_5m	3	5	30.084	−132.080
DCM (108–130 m)	S4_108m	3	108	30.084	−132.080
Below DCM (175–200 m)	S4_180m	3	180	30.084	−132.080
Mesopelagic (500 m)	S4_500m	3	500	30.084	−132.080
Surface (5 m)	S5_5m	2	5	29.918	−132.082
DCM (108–130 m)	S5_110m	2	110	29.918	−132.082
Below DCM (175–200 m)	S5_200m	2	200	29.918	−132.082
Mesopelagic (500 m)	S5_500m	2	500	29.918	−132.082

### 2.2 DNA Extraction and Amplicon Sequencing

As previously described (Semedo et al. 2021), total DNA was extracted from the Sterivex filters using the DNeasy PowerWater Sterivex DNA Isolation Kit protocol (Qiagen), following manufacturer's instructions. The 16S rRNA gene was amplified in preparation for both short‐reads (Illumina) and long‐reads (PacBio) sequencing.

For Illumina sequencing, the 16S rRNA gene was amplified with the degenerate primer pair 515YF (5′‐GTGYCAGCMGCCGCGGTAA‐3′) and Y926R‐jed (5′‐CCGYCAATTYMTTTRAGTTT‐3′), targeting the hypervariable V4‐V5 region, covering a broad spectrum of marine microbial diversity (Apprill et al. 2015; Parada, Needham, and Fuhrman 2016) as well as high internal diversity within the *Planctomycetota* phylum (Fadeev et al. 2021). The initial PCR reaction included 12.5 ng of template DNA in a total volume of 25 μL. The PCR protocol involved a 3 min denaturation step, followed by 25 cycles of 98°C for 20 s, 60°C for 30 s and 72°C for 30 s, and, finally, an extension stage at 72°C for 5 min. A second PCR reaction was performed to add indexes and sequencing adapters to the target region, according to manufacturer's recommendations (https://www.illumina.com/). Negative controls without template were included in all PCR reactions. Lastly, PCR products were one‐step purified and normalised using SequalPrep 96‐well plate kit (ThermoFisher Scientific, Waltham, USA), pooled, and pair‐end sequenced in the Illumina MiSeq sequencer using 2 × 300 bp with the V3 chemistry, according to manufacturer instructions (Illumina, San Diego, CA, USA) at Genoinseq (Cantanhede, Portugal).

For PacBio sequencing, the 16S rRNA gene was amplified with the degenerate primer pair 27F (5′‐AGRGTTYGATYMTGGCTCAG‐3′) and 1492R (5′‐RGYTACCTTGTTACGACTT‐3′), targeting the full 16S rRNA gene, theoretically providing increased phylogenetic resolution due to the coverage of several hypervariable regions (Lane 1991; Paliy et al. 2009). Amplicon fragments were previously PCR‐amplified from the DNA in duplicate using separate template dilutions using the high‐fidelity Phusion Plus polymerase. A single round of PCR was performed using full‐length 16S rRNA primers. PCR products were visually verified by running on a high‐throughput Hamilton Nimbus Select robot using Coastal Genomics Analytical Gels. The PCR reactions from the same samples were then pooled in one plate, cleaned‐up and normalised using the high‐throughput Charm Biotech Just‐a‐Plate 96‐well Normalisation Kit. PacBio samples were then pooled to make one library which was quantified fluorometrically before sequencing with PacBio Sequel 2 platform.

The results from 16S rRNA gene amplicon long‐reads and short‐reads sequencing are publicly available in the ENA‐EMBL archive with the project accession number PRJEB32783. Primers used in this study for 16S rRNA gene amplification have been tested in an in silico PCR against other primers present in the literature for the study of *Planctomycetota* phylum. Such primer sets were 515YF/Y926R‐jed (Apprill et al. 2015; Parada, Needham, and Fuhrman 2016), 27F/1429R (Lane 1991; Paliy et al. 2009), used in the present study; PLA46F/1542R (Bengtsson and Øvreås 2010; Kirkpatrick et al. 2006), 58F/926R (Kirkpatrick et al. 2006) and PLA352F/PLA920R (Mühling et al. 2008). These five primer sets were tested with (i) SILVA TestPrime (Klindworth et al. 2013) and with (ii) a in silico PCR using a manually‐curated set of reference genomes using an ad‐hoc Python script. For the in silico PCR with the manual curated data, a reference dataset of genomes composed of 130 *Planctomycetota* was used. The number of matches of such primers were counted for reference genome, together with the total number of matches in the dataset per pair primers. Results are available in Tables S4, S5, and S6.

### 2.3 Bioinformatic Analysis

Samples were sequenced both in Illumina and PacBio platforms producing paired‐end (PE) 300 bp short‐reads and circular‐consensus sequencing (CCS) long‐reads, respectively.

For Illumina, 16S rRNA gene sequences were imported into QIIME2 (Bolyen et al. 2019) and DADA2 (Callahan et al. 2016) was run with the specific option for PE short‐reads. Forward and reverse reads were truncated at 280 and 270 bp, respectively, to keep only bases with an average Phred quality score above 30. A table with the complete DADA2 statistics is available in Table S1. ASVs resulting from the analysis were then classified using a scikit‐learn Naive‐Bayes classifier trained on the SILVA database v. 138.1 (Yilmaz et al. 2014). The choice of SILVA resulted from a preliminary analysis where SILVA and Genome Taxonomy Database (GTDB) were compared on the ability to identify our desired target (*Planctomycetota* members). The ASVs from the taxonomic table were then filtered, selecting only ASVs belonging to the *Planctomycetota* phylum. Biodiversity indexes, such as alpha and beta diversities, were calculated using the Python libraries scikit‐bio (https://scikit.bio) and scipy (https://scipy.org). In particular, alpha diversity has been calculated using Shannon index and observed features index. Beta diversity has been calculated using Bray–Curtis dissimilarity and clustered using single linkage, namely the Nearest Point algorithm to produce a dendrogram where each cluster is composed by drawing a U‐shaped link between a non‐singleton cluster and its children.

For PacBio, 16S rRNA gene sequences were imported into QIIME2 (Bolyen et al. 2019) as well as DADA2 (Callahan et al. 2016) was run with the specific option for circular consensus sequencing (CCS) reads. Only reads with a length between 1000 and 1600 bp were kept. A table with the complete DADA2 statistics is available in Table S2. ASVs resulting from the analysis were then classified using a scikit‐learn Naive‐Bayes classifier trained on the same SILVA database of the Illumina reads, for consistency. The ASVs from the taxonomic table classified ASVs were then filtered, selecting only ASVs belonging to the *Planctomycetota* phylum. Biodiversity indexes were calculated with the same methodology used for Illumina reads. The use of two different sequencing platforms with different read lengths allowed us to describe eventual differences between technologies and evaluate possible advantages of one of the approaches for the study of *Planctomycetota*.

Differences in the alpha diversity measures obtained in the different investigated layers were assessed through a statistical analysis composed of a series of Wilcoxon tests. In particular, it was assessed if (i) PacBio and Illumina resulted in different alpha diversity and (ii) if PacBio alpha diversity was greater than the one obtained with Illumina data. This consisted in 2 Wilcoxon signed‐rank test for hypothesis (i) (one for Shannon metric, one for observed ASVs metric) and 2 Wilcoxon signed‐rank test for hypothesis (ii) (as before, one for Shannon metric, one for observed ASVs metric). Differences in the beta diversity measures obtained in the different investigated layers were assessed with a PERMANOVA test.

The phylogeny of the *Planctomycetota* ASVs obtained from both PacBio CCS and Illumina reads was also manually curated to improve taxonomic assignment accuracy within our target phylum. This was necessary due to taxonomic inconsistencies observed when analysing the SILVA outputs, mainly at lower taxonomic levels. This was achieved by direct comparison of the 16S rRNA gene sequences from this study with the up to date publicly available sequence data on *Planctomycetota* type strains (taken from the National Center for Biotechnology Information (NCBI) database) using the nucleotide Basic Local Alignment Search Tool (BLAST) available in NCBI. The taxonomic inference was done by using well‐established thresholds for delineation of prokaryotic taxa (98.7%, 94.5%, 86.5%, 82.0% and 78.5% 16S rRNA gene similarity for species, genus, family, order and class, respectively) (Yarza et al. 2014). To represent the *Planctomycetota* ASVs in the phylum, the sequences from PacBio CCS were aligned with the ones from *Planctomycetota* type strains using CLUSTALW (Larkin et al. 2007) and a maximum likelihood phylogenetic tree constructed using the MEGAX software, the general time reversible model and the activated gamma distributed with invariant sites (G + I) option (Kumar et al. 2018). iTOL was then used for tree annotation (Letunic and Bork 2021).

## 3 Results

### 3.1 Physicochemical Structure of the Sampling Layers

The physicochemical characterisation of the various sampling zones was previously published (Semedo et al. 2021) and is shown in Table S3. Briefly, surface waters at the sampling location have higher levels of temperature and dissolved oxygen, very low fluorescence levels and low NO_2_^−^, NO_3_^−^ and NH_4_^+^, when compared to deeper layers. The presence of a photosynthetic microbial community, phytoplankton, in the DCM zone is responsible for significantly greater fluorescence values. As a result, dissolved O_2_ levels are high, and a temperature drop of roughly 3°C in DCM relative to the surface. In the Below DCM layer, there was an 8°C temperature decrease in comparison to the surface, as well as a decrease in dissolved O_2_ and a significant increase in NO_3_^−^ levels (a 753‐fold increase). In the mesopelagic layer, temperature and dissolved O_2_ declined (13.4°C and 135.4 μM, respectively), but NO_3_^−^ levels increased dramatically (2444 fold).

### 3.2 Primers Testing and Comparison

The different primer sets tested showed great variability in the ability to retrieve *Planctomycetota*. SILVA TestPrime results showed that the pair 27F/1492R and 515F/Y926R‐jed were the most successful, with 69% and 85% of *Planctomycetota* matches, respectively (Table S5). The in silico PCR analysis of curated reference *Planctomycetota* genomes confirmed previous results from SILVA PrimeTest, identifying 27F/1492R and 515F/Y926R‐jed as the most suitable primers for studying cultured *Planctomycetota*. These primers matched 59.69% and 57.36% of the *Planctomycetota* genomes, respectively (Table S6). The primers that were used in this paper proved to be suitable for the study of *Planctomycetota*.

### 3.3 Amplicon Sequencing Overview

The comparison between Illumina and PacBio sequencing revealed differences in sequence output, quality and ASV calling. These differences are important as sequencing output strongly influences the following step of the analysis. In Illumina, sequencing resulted in an average number of 61,495 sequences per sample, with a mean quality of 30 (Phred score range from 0 to 62) and a length of 282 nucleotides. In PacBio, sequencing resulted in an average number of 21,778 sequences per sample, with a mean quality of 80 (Phred score range from 0 to 93) and a median length of 1457 nucleotides. ASVs calling resulted in a total of 6379 and 3252 ASVs with PacBio and Illumina sequencing, respectively (Table 2). In total, 154 ASVs were classified as *Planctomycetota* with Illumina, representing 4.8% of the total number of ASVs. PacBio classified 251 *Planctomycetota* ASVs, representing 3.9% of the total number of ASVs. Rarefaction curves have been calculated (Figure S2), showing that curves reached a plateau in both sequencing platforms. This indicates that our findings accurately represent the microbial diversity within the samples, obtained with the sets of primers used.

**TABLE 2:** Comparison of the phylogenetic *Planctomycetota* distribution between PacBio and Illumina methodologies. In each taxonomic division (phylum, classes, orders, families and genera), percentages of ASVs for each group were calculated.

		PacBio		Illumina	
		Number of ASVs	%	Number of ASVs	%
Phylum	*Planctomycetota*	251	3.9	154	4.8
	Other phyla	6128	96.1	3098	95.2
Class/lineage	*Planctomycetia*	78	31.1	61	39.6
	*Phycisphaerae*	76	30.3	36	23.4
	Anammox (‘*Candidatus* Brocadiia’)	25	10.0	24	15.6
	OM190 lineage/‘Saltatorellus’ clade	72	28.7	31	20.1
	Unknown	0	0	2	1.3
Order	*Pirellulales*	44	17.5	42	27.3
	*Planctomycetales*	34	13.5	17	11.0
	*Gemmatales*	0	0	1	0.6
	*Phycisphaerales*	74	29.5	33	21.4
	*Sedimentisphaerales*	2	0.8	3	1.9
	*CA* Brocadiales	24	9.6	22	14.3
	OM190 lineage/‘Saltatorellus’ clade	72	28.7	31	20.1
	Unknown	1	0.4	5	3.2
Family	*Lacipirellulaceae*	7	2.8	10	6.5
	*Pirellulaceae*	35	13.9	26	16.9
	*Planctomycetaceae*	33	13.1	16	10.4
	*Thermoguttaceae*	0	0	1	0.6
	*Phycisphaeraceae*	39	15.5	12	7.8
	*Anaerohalosphaeraceae*	0	0	2	1.3
	*CA* Brocadiaceae	1	0.4	7	4.5
	OM190 lineage/‘Saltatorellus’ clade	72	28.7	31	20.1
	Unknown	64	25.5	49	31.8
Genus	Known	0	0	5	3.2
	Unknown	251	100	149	96.8

Abbreviation: *CA*, *Candidatus*.

### 3.4 *Planctomycetota* Diversity

**FIGURE 1:**
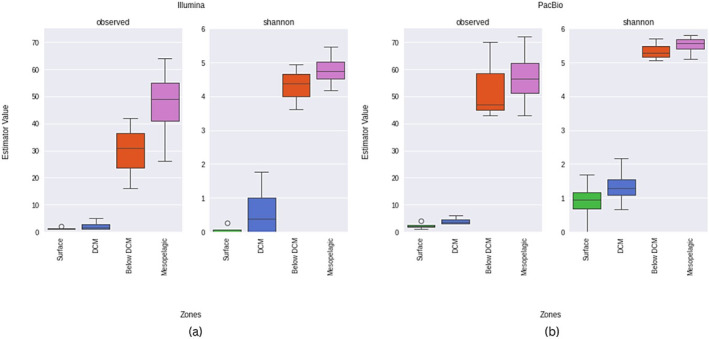
*Planctomycetota* alpha diversity across the different depths. Boxplots show alpha diversity calculated with two different metrics, observed ASVs and Shannon. Samples were grouped according to the four water column depth layers considered (Surface, DCM, Below DCM, and Mesopelagic). In (a) results using reads obtained from Illumina sequencing while in (b) reads were obtained from PacBio platform.

Alpha diversity analysis resulted in the increasing *Planctomycetota* diversity in the water column with depth (Figure 1). Clearly, lower alpha diversity values were registered in the surface and DCM layers, compared with below DCM and the mesopelagic layers. These differences are consistent across the Illumina and PacBio sequencing platforms.

Despite such consistency between platforms, it is important to notice that alpha diversity metrics differ in their values between short and long 16S rRNA reads approaches. Results suggested that the PacBio approach was able to recover higher *Planctomycetota* diversity at the DCM and Mesopelagic layers, especially for observed ASVs in the below DCM layer as well as for Shannon estimator in the DCM and below DCM layers, with higher values detected with the PacBio platform (Figure 1). This was tested through a Wilcoxon test, to evaluate if (i) the differences between alpha diversity measures were statistically significant between different platforms and if (ii) PacBio diversity was greater than the one obtained from Illumina. It resulted that both diversity metrics (Shannon and observed ASVs) were different between PacBio and Illumina (*p*‐value = 0.00061 for Shannon, *p*‐value = 0.012451 for observed ASVs). Moreover, PacBio diversity showed to be greater than the one obtained from Illumina (*p*‐value = 0.000305 for Shannon, *p*‐value = 0.006226 for observed ASVs).

**FIGURE 2:**
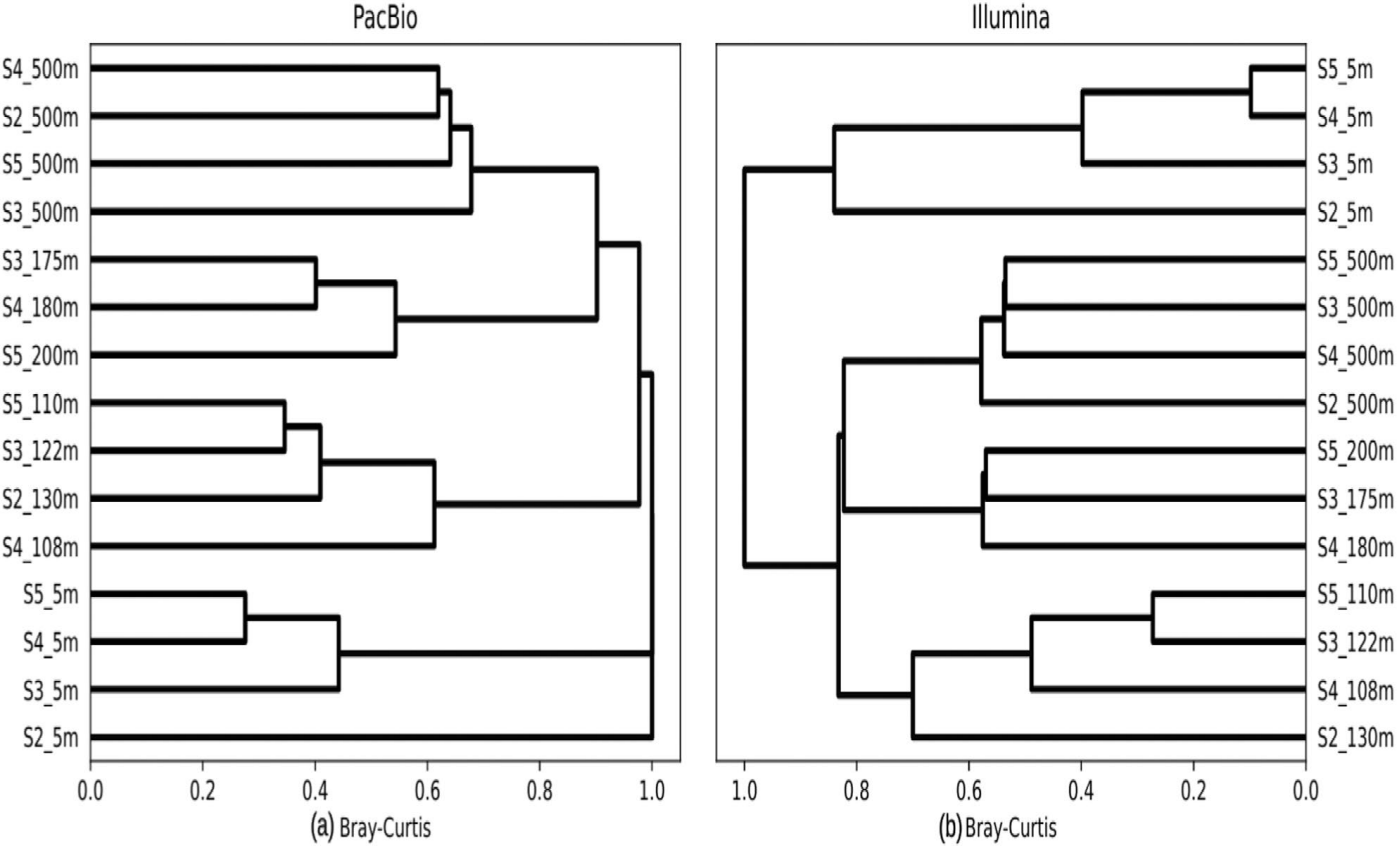
*Planctomycetota* beta‐diversity clustering with different sequencing platforms, PacBio (a) and Illumina (b). Samples clustered according to depth layers.

The beta diversity analysis revealed a clustering pattern of the *Planctomycetota* communities based on the water column depth (Figure 2). Differences between the clusters generated were confirmed as significant with a PERMANOVA test for both Illumina and PacBio data, resulting in a *p*‐value of 0.001 for both. These findings underscore the significant diversity of environmental conditions within the examined water column resulting in a selection of different *Planctomycetota* communities. The two sequencing platforms consistently showed that surface samples were more dissimilar with respect to *Planctomycetota* community structure present in the other layers. Additionally, communities in the deeper layers (Below DCM and Mesopelagic) showed greater similarities to each other than to those in the surface and DCM layers. Besides the degree of community dissimilarity between the different water column layers, beta diversity analysis showed that samples from the different water column depths cluster independently. This indicates that the water column environmental gradients are reflected in the *Planctomycetota* community structure, where the communities present in different water layers are significantly different between each other.

### 3.5 *Planctomycetota* Community Composition

**FIGURE 3:**
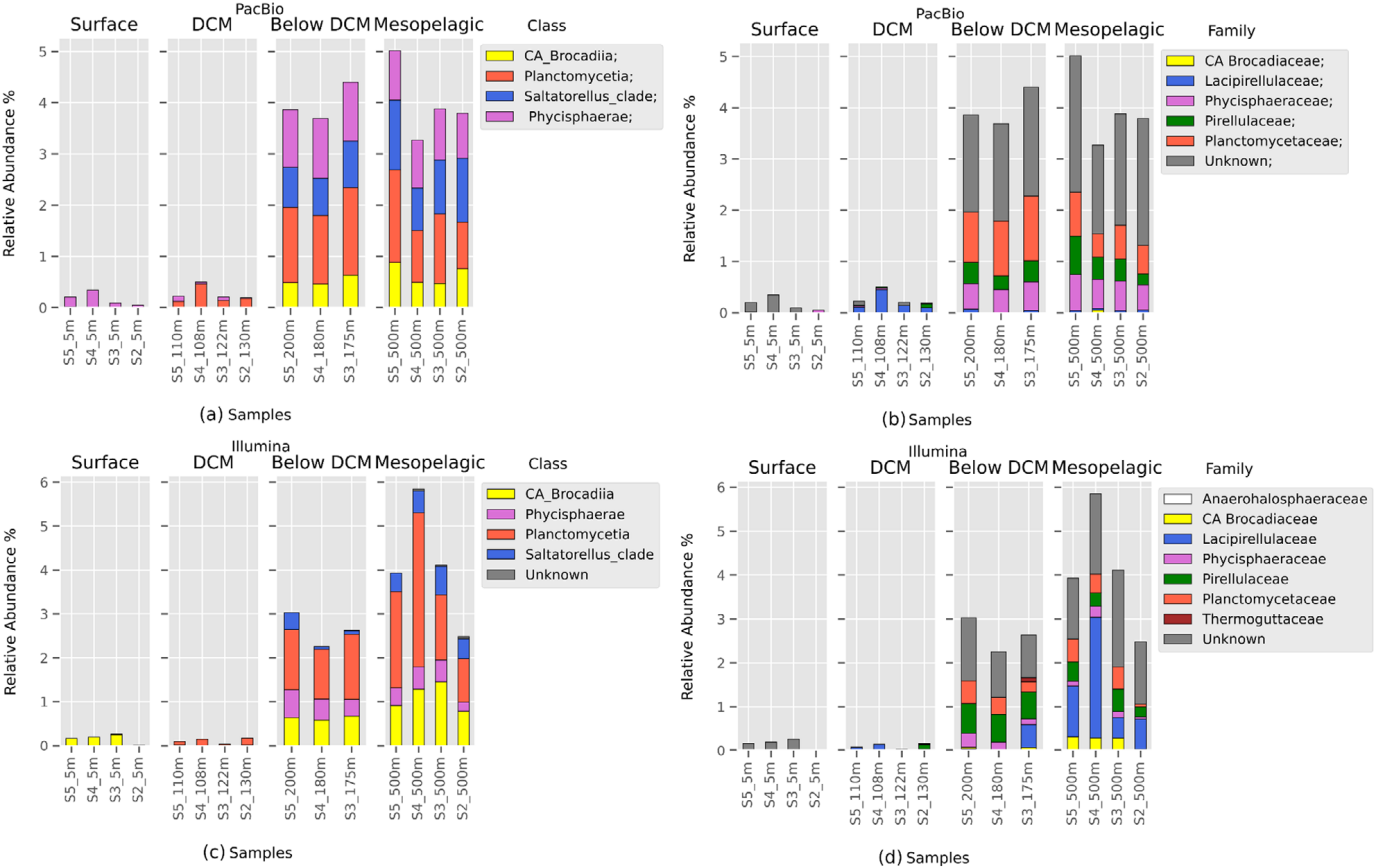
*Planctomycetota* community composition for the four investigated layers based on results from PacBio platform (a and b) and from Illumina platform (c and d). In (a and c)the community was studied at Class level while in (b and d) at Family level. Relative abundance was calculated by dividing the reads assigned to each *Planctomycetota* taxa by the total number of reads in each sample and then multiplied by 100 to obtain a percentage.

The *Planctomycetota* community composition at all taxonomic levels is shown in Table 2 (except species level due to inexistence of taxa at this taxonomic level). Interestingly, all 251 *Planctomycetota* ASVs found through PacBio sequencing were unclassified or unknown at the genus level. Additionally, out of 154 *Planctomycetota* ASVs found through Illumina sequencing, 149 (96.8%) were unclassified or unknown at genus level. Community composition was further investigated at the Class and Family levels based on the different sequencing platform data, due to the impossibility of classifying any ASVs at Genus or Species level. PacBio and Illumina showed a consistent pattern where relative abundance of *Planctomycetota* increased significantly in Below DCM and Mesopelagic layers. Four classes were identified: *Planctomycetia*, *Phycisphaerae*, ‘*Candidatus* Brocadiia’ and ‘*Saltatorellus*’ clade, with both platforms (Figure 3).

The relative abundance of obtained classes was different according to the sequencing platform of choice. For example, *Saltatorellus* clade and *Phycisphaerae* were more abundant in the community based on PacBio than based on Illumina. An evident discrepancy between the two platforms is at the Surface layer where Illumina attribute the class ‘*Candidatus* Brocadiia’ as the most abundant in such a layer while PacBio refers to the *Phycisphaerae* class. Additionally, Illumina platform presented *Planctomycetota* ASVs that were not classified at the Class level in the Mesopelagic layer, while in the PacBio dataset all *Planctomycetota* classes were classified. Furthermore, the difference between the Below DCM layer and Mesopelagic layer was more pronounced in Illumina data with respect to the PacBio. Differences were shown also regarding the proportion of the single classes/families inside a sample. For example, in sample 9 short reads sequencing reports the class *Planctomycetia* to be predominant, while long reads shows a similar relative abundance of the class detected. At family level, ASVs were classified in five families in PacBio and in seven families in Illumina. The five *Planctomycetota* families detected by Pacbio (‘*Candidatus* Brocadiaceae’, *Lacipirellulaceae*, *Phycisphaeraceae*, *Pirellulaceae*, *Planctomycetaceae*) were also detected by Illumina, while *Thermoguttaceae* and *Anaerohalosphareaceae* were exclusively detected by the latter. In the two deeper water column layers (Below DCM and Mesopelagic), long reads showed a higher relative abundance of unknown ASVs at the family level (average 1.05%), when compared to the Illumina short‐reads (average 0.73%).

### 3.6 *Planctomycetota* Phylogeny

**FIGURE 4:**
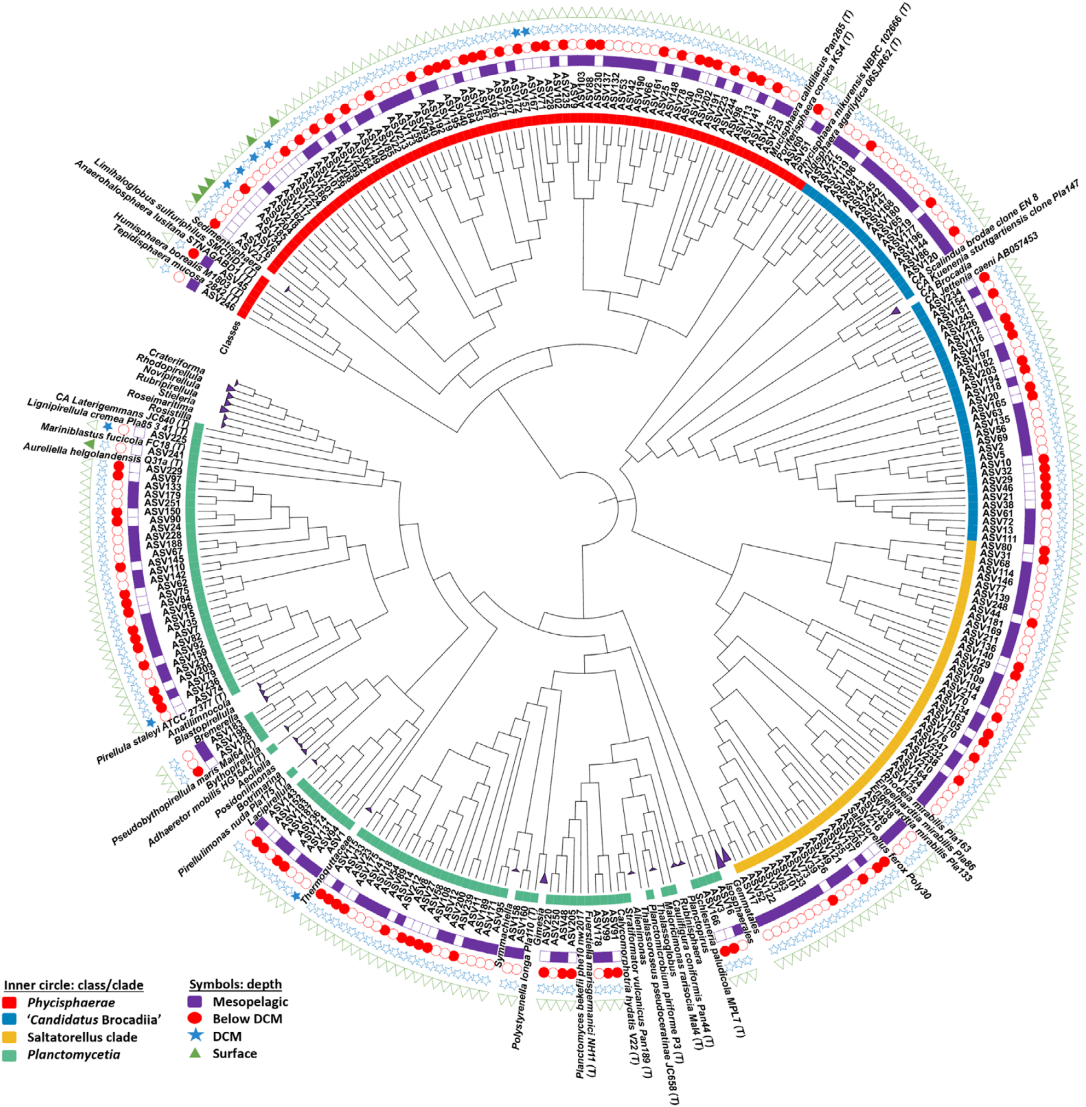
Maximum likelihood phylogenetic tree showing the taxonomy/phylogeny of the planctomycetotal ASVs obtained using PacBio sequencing. 16S rRNA gene sequences of *Planctomycetota* type strains (taken from the NCBI database) were added to the tree for comparison. The inner circle represents the class/clade inside the phylum *Planctomycetota* and, the outer symbols, the depths where each ASV was found.

Using the long sequencing generated through PacBio, a manually curated phylogenetic analysis of the ASVs assigned to the *Planctomycetota* phylum and all described type strains was performed, resulting in the tree presented in Figure 4. PacBio sequences were chosen for their greater length, which allowed a more precise alignment and tree construction. ASVs were not phylogenetic linked to any of the known described species but rather grouping in unknown clusters. This exciting result indicates that in the open ocean of the area of study the diversity of *Planctomycetota* is entirely unknown. Unrelated branches resulted distantly related within the *Phycisphaerae*, the ‘*Candidatus* Brocadiia’ (anammox), the OM190—‘Saltatorellus’ clade and to a lesser extent within the *Planctomycetia*, the class with the highest number of described species (Vitorino and Lage 2022). In line with the previously presented results (Figures 1 and 3), *Planctomycetota* were rare at the surface waters and in the DCM layer and increased representativeness Below DMC and in the mesopelagic zones (Figure 4). Overall, higher number of planctomycetotal ASVs are affiliated to the class *Planctomycetia* (31.1%) followed by *Phycisphaerae* (30.3%), OM190 lineage/’Saltatorellus’ clade (28.7%) and finally by the ‘*Candidatus* Brocadiia’ (10%), the anammox group within this phylum (Table 2).

The most diverse *Planctomycetota* ASVs were present in the below DCM and Mesopelagic layers (Figure 4), but the relatively most abundant ASV was detected in the DCM layer, and related to the family *Lacipirellulaceae*. The phylogenetic classification and distribution of the *Planctomycetota* ASVs generally agree between the two methods, especially at higher taxonomic levels (Table 2 and Figures 3 and 4). According to the phylogenetic analyses performed, no ASV could be classified at the species level with either of the two methodologies, which emphasises the novelty of the *Planctomycetota* in this habitat.

## 4 Discussion

In this study, *Planctomycetota* represented 4.8% (Illumina) or 3.9% (PacBio) of the total bacterial number of ASVs. *Planctomycetota* are generally represented in low numbers in marine pelagic environments. Similar percentages of *Planctomycetota* representatives were observed by Sousa and collaborators (de Sousa et al. 2019) in Arctic seawater collected under snow‐covered sea ice at different water column depths (5, 20 and 50 m), and in seawater samples collected from the meso‐ and bathypelagic water masses in the dark western Mediterranean (Mena et al. 2021).

Our results show how the *Planctomycetota* distribution is well stratified in the open ocean water column, in terms of diversity and community structure presenting a clear distinction between the two upper (surface and DCM), and the two lower (below DCM and mesopelagic) water column layers. These microorganisms demonstrate a preference for deeper ocean habitats, where both their relative abundance and community diversity are notably higher compared to shallower depths. This preference reflects the diverse ecosystems that emerge across the ocean's water layers, each distinguished by unique environmental conditions (Table S3). The surface waters were poor in inorganic nutrients (NO_2_^−^, NO_3_^−^ and NH_4_^+^), and the presence of phytoplankton in the deep chlorophyll maximum (DCM) zone boosts oxygen production (Semedo et al. 2021). In the nelow DCM layer, there was an 8°C temperature decrease in comparison to the surface, as well as a decrease in dissolved O_2_ and a significant increase in NO_3_^−^ levels (a 753‐fold increase) (Semedo et al. 2021). The deeper layer (mesopelagic) experience even lower oxygen and temperatures (a decline of 13.4°C in temperature and 135.4 μM of O_2_ levels relatively to surface), with a corresponding rise in nitrate levels (2444 fold) (Semedo et al. 2021). The water column environmental profile creates distinct ecological niches shaping the abundance and diversity of *Planctomycetota* in each layer.

With the exception of the anaerobic ammonium oxidation (anammox) bacteria (‘*Candidatus* Brocadiia’) that are chemotrophs, most of the identified *Planctomycetota* are known heterotrophs (Lage et al. 2019). In the euphotic DCM layer, organic matter is produced by the phytoplankton and, after sinking to the dark zones, it feeds the microbial community there (Bergauer et al. 2018; Kirchman 2018). In the oceans, heterotrophic bacteria use extracellular enzymes to break down large substrates like polysaccharides into small‐sized molecules (Giljan et al. 2023) and play a significant role in the global carbon cycle. As previously described (DeLong, Franks, and Alldredge 1993; Pizzetti et al. 2011; Suter et al. 2018; Thompson, Valentine, and Peng 2024), *Planctomycetota* are mainly associated in aggregates in marine snow, benefiting from the organic matter produced in the euphotic zone by the autotrophic microbial community. Furthermore, these two zones (below DCM and Mesopelagic) have abundance of members from the archaeal phylum *Thaumarchaeota* (syn. *Nitrososphaerota*) (ammonium oxidising archaea—AOA) and the bacterial phylum *Nitrospinota* (nitrite oxidising bacteria—NOB) that convert ammonium into nitrite and nitrite into nitrate, respectively (Semedo et al. 2021). This may contribute to the higher levels of nitrate in these zones, which are an important source of nitrogen for the *Planctomycetota*.

*Planctomycetota* in deeper layers may also benefit from feeding on large molecules potentially using a ‘selfish’ behaviour. The so‐called ‘selfish’ bacteria are bacteria that use uptake mechanisms of larger oligosaccharides keeping substantial quantities of substrate in the periplasmic space (Cuskin et al. 2015). Reintjes et al. (2017) demonstrated that, in the Atlantic Ocean, 26% of total cells carry out ‘selfish’ uptake of specific polysaccharides. Through fluorescence in situ hybridization (FISH) and uptake of fluorescently labelled polysaccharides, Reintjes and collaborators (Reintjes et al. 2019) and Giljan and collaborators (Giljan et al. 2022) showed that *Planctomycetota* show this ‘selfish’ behaviour. *Planctomycetota* are well known for their outstanding hydrolytic potential, namely of complex polysaccharides degradation: they possess an exceptionally high numbers of sulfatase genes (Wegner et al. 2014; Faria et al. 2018) and a high number of carbohydrate‐active enzymes (CAZymes) encoded in their genomes (Dedysh and Ivanova 2019; Klimek, Herold, and Calusinska 2024). Several studies demonstrated the capacity of different *Planctomycetota* for macromolecules degradation (Dedysh and Ivanova 2019; Cutts et al. 2022). Woebken et al. (2007) had already proposed the entrapment of sulfated polysaccharides in the commonly *Planctomycetota* that colonise marine snow aggregates. Besides the referred capacities, *Planctomycetota* also possess other characteristics that favours the ‘selfish’ uptake. These include (i) an enlarged periplasm (Boedeker et al. 2017), (ii) pili that were suggested to take part in the uptake of complex polysaccharides like dextran (Boedeker et al. 2017), (iii) endocytosis‐like macromolecule uptake (Boedeker et al. 2017), (iv) the presence of ‘planctosomes’ structures that are comparable to cellulosomes, which are extracellular protein complexes involved in the degradation of plant cell wall‐derived polysaccharides (Andrei et al. 2019) or of ‘bacterial microcompartments’ involved in the degradation of plant and algal cell wall sugars such as L‐fucose and L‐rhamnose (Erbilgin, McDonald, and Kerfeld 2014) and (v) giant genes encoding putative polysaccharide catabolic enzymes (e.g., pectate lyases and pectinesterases) (Kallscheuer and Jogler 2021).

Anammox bacteria have the unique metabolic ability to combine ammonium and nitrite or nitrate to form nitrogen gas (van Niftrik and Jetten 2012). Members of these bacteria (‘*Candidatus* Brocadiia’) were present only in the below DCM and in the mesopelagic layers, probably living in aggregates where the O_2_ level were even lower than the detected values for these layers (mean values of 4.62 and 0.86 mg L^−1^ respectively for below DCM and mesopelagic layers; Table S3) (Brück et al. 2010; Lehto et al. 2014; Wessel et al. 2014). The ‘*Candidatus* Brocadiia’ presence in the surface layer, detected with Illumina sequencing, is not clear and may be the result of a misassignment of the ASVs as anammox bacteria are usually located in the anoxic oceanic area, and their contribution in various oceanic areas is considerably different (Kuypers et al. 2003; Wei and Zhang 2023). Alternatively, it could represent the occurrence of sub‐oxic microhabitats in a generally oxic water column layer (Ploug et al. 1997; Bianchi et al. 2018) or by mixing of water masses with dormant bacteria. As it can be seen in Table S3, in deeper layers, particularly in samples S3_175m (below DCM) and S5_500m (mesopelagic), where the nitrite levels (0.038 and 0.155 μM, respectively) were higher comparatively to the upper layers, and in S4_500m and S2_500m (both mesopelagic samples), where ammonium levels were the highest in our samples (0.11 and 0.65 μM, respectively), contained higher occurrences of anammox *Planctomycetota*.

Members of the recently proposed ‘Saltatorellus’ clade (Wiegand et al. 2020) were found mainly in the mesopelagic layer (Figure 3). This clade has long been found in environmental samples and referred to as OM190 group. OM190 was first described in a study where the role of bacterioplankton community structure on organic carbon transformations was analysed in the eastern continental shelf of the United States near Cape Hatteras, North Carolina (Rappé, Kemp, and Giovannoni 1997). Presently in the Silva database, there are 12,148 entrances for OM190 and all these are still considered within the *Planctomycetota*. In fact, they have been found in the microbial community of macroalgal biofilm (Bengtsson and Øvreås 2010; Bengtsson et al. 2012; Lage and Bondoso 2014; Bondoso et al. 2017), associated with *Bacillariophyta* (Pushpakumara et al. 2023), in the oxygenated hypolimnion of lakes (Okazaki et al. 2017; Storesund et al. 2020), in marine and freshwater sediments (Storesund and Øvreås 2013; Andrei et al. 2019) and associated with particles in marine waters (Bizic‐Ionescu et al. 2015; Salazar et al. 2015; Thompson, Valentine, and Peng 2024). In the meromictic Lake Sælenvannet, they were the most abundant group of *Planctomycetota* in the transition zone between oxic and anoxic conditions (Storesund et al. 2020). In our study, they were also observed in the O_2_ transition zone.

In this work, we used two sequencing methodologies to assess the planctomycetotal community present in one transect in the Pacific Ocean: short‐reads using Illumina platform and long‐reads using PacBio. These approaches agreed both on the diversity and stratification of the *Planctomycetota* community. However, PacBio data resulted in higher alpha diversity values and higher number of *Planctomycetota* ASVs (251 against the 154 obtained with Illumina, Table 2). Moreover, the length of the reads produced with PacBio allows a greater phylogenetic resolution than Illumina (Johnson et al. 2019). Additionally, the full‐length 16S rRNA gene sequence contains all the hypervariable regions present in the gene, while with Illumina only V4 and V5 regions have been targeted. Despite these differences, we do not have enough information to define whether one technique should be preferentially used in respect to the other for the study of *Planctomycetota* communities. However, the situation is different when a phylogenetic analysis is intended to be performed. It is known that using longer sequences allows for more robust phylogenetic inference by providing greater phylogenetic signal, reducing the impact of rate variation, and mitigating long‐branch attraction issues, ultimately leading to more accurate phylogenetic trees (Pollock et al. 2002; Philippe et al. 2011). This would lead to the preference of PacBio sequencing due to its ability of producing longer reads.

The study of microbial communities using amplicon‐based data is significantly influenced by primer selection. Previous research has described the use of specific primers for targeting the *Planctomycetota* phylum (Bengtsson and Øvreås 2010; Kirkpatrick et al. 2006; Mühling et al. 2008). Despite the substantial advancements in understanding the *Planctomycetota* due to targeted studies, certain groups within this phylum may remain undetectable with these specific primers. For instance, McNichol et al. (2021) did not detect *Planctomycetota* in samples from the Atlantic and Pacific Oceans using their chosen primers.

In contrast, the primers utilised in the present study successfully detected a significant number of *Planctomycetota*, including several novel taxa. These primers are widely applied in microbial environmental analyses, enhancing the comparability of results across different studies. Additionally, Thompson, Valentine, and Peng (2024) detected substantial levels of *Planctomycetota* in samples from the Eastern Tropical North Pacific Ocean using non‐*Planctomycetota*‐targeted primers. This is in agreement with the in silico evaluation comparing the primers used in the current study with *Planctomycetota*‐specific primers demonstrated that the 27F/1492R and 515F/Y926R‐jed primers yielded a higher detection percentage of *Planctomycetota*.

## 5 Conclusions

This study indicates that *Planctomycetota* inhabiting oceanic environments represent an untapped and previously uncharacterized reservoir of novel bacteria within this phylum, exhibiting uneven distribution throughout the water column. The fact that none of the ASVs generated from both Illumina and PacBio data were identified at species levels highlights the potential of discovering novel organisms in the studied environment. Our data reveal that these organisms prefer the depths below the deep chlorophyll maximum (DCM) zone, where high levels of nitrate are present, providing essential nutrients for the growth of heterotrophic bacteria. Consistency between different sequencing platforms confidently increased the reliability of our findings, despite the long reads obtained with PacBio sequencing offering a significant advantage for studying their phylogeny. Future works may explore these novel species to broaden our understanding of *Planctomycetoata* phylum in the Pacific Ocean.

## Author Contributions

**Inês Rosado Vitorino:** data curation, formal analysis, visualization, writing – original draft, writing – review and editing. **Nicola Gambardella:** data curation, formal analysis, visualization, writing – original draft, writing – review and editing. **Miguel Semedo:** formal analysis, writing – original draft, writing – review and editing, investigation. **Catarina Magalhães:** conceptualization, writing – review and editing, writing – original draft, funding acquisition, investigation, methodology, sampling, supervision. **Olga Maria Lage:** conceptualization, formal analysis, writing – original draft, writing – review and editing, supervision.

## Conflicts of Interest

The authors declare no conflicts of interest.

## Supporting information


Data S1.

## Data Availability

All sequencing data produced in this study is publicly available in the ENA‐EMBL archive (project accession number: PRJEB32783). The CTD dataset is publicly available in PANGAEA (https://doi.pangaea.de/10.1594/PANGAEA.903405).
