# Supplementary material for: Diversity and Vertical Distribution of Planctomycetota in the Water Column of the Remote North Pacific
Source: Environ Microbiol Rep. 2025 Feb 20;17(1):e70063. doi: 10.1111/1758-2229.70063 (PMC11840708; doi:10.1111/1758-2229.70063)
Supplement: Supplementary file 1 — Data S1. [file EMI4-17-e70063-s001.docx]

**Diversity and depth distribution of *Planctomycetota* in water column of the remote North Pacific**

^*^Inês Rosado Vitorino^1,2^, ^*^Nicola Gambardella^2^, Miguel Semedo^2^, Catarina Magalhães^1,2^, Olga Maria Lage^1,2^

*These authors contributed equally to this work

^1^Department of Biology, Faculty of Sciences and CIIMAR, University of Porto, Portugal

^2^ Interdisciplinary Centre of Marine and Environmental Research (CIIMAR/CIMAR), University of Porto, Porto, Portugal

Corresponding authors: Catarina Magalhães - catarina.magalhaes@fc.up.pt and Olga Maria Lage - olga.lage@fc.up.pt

**Supporting information**

**Supplementary Figures**


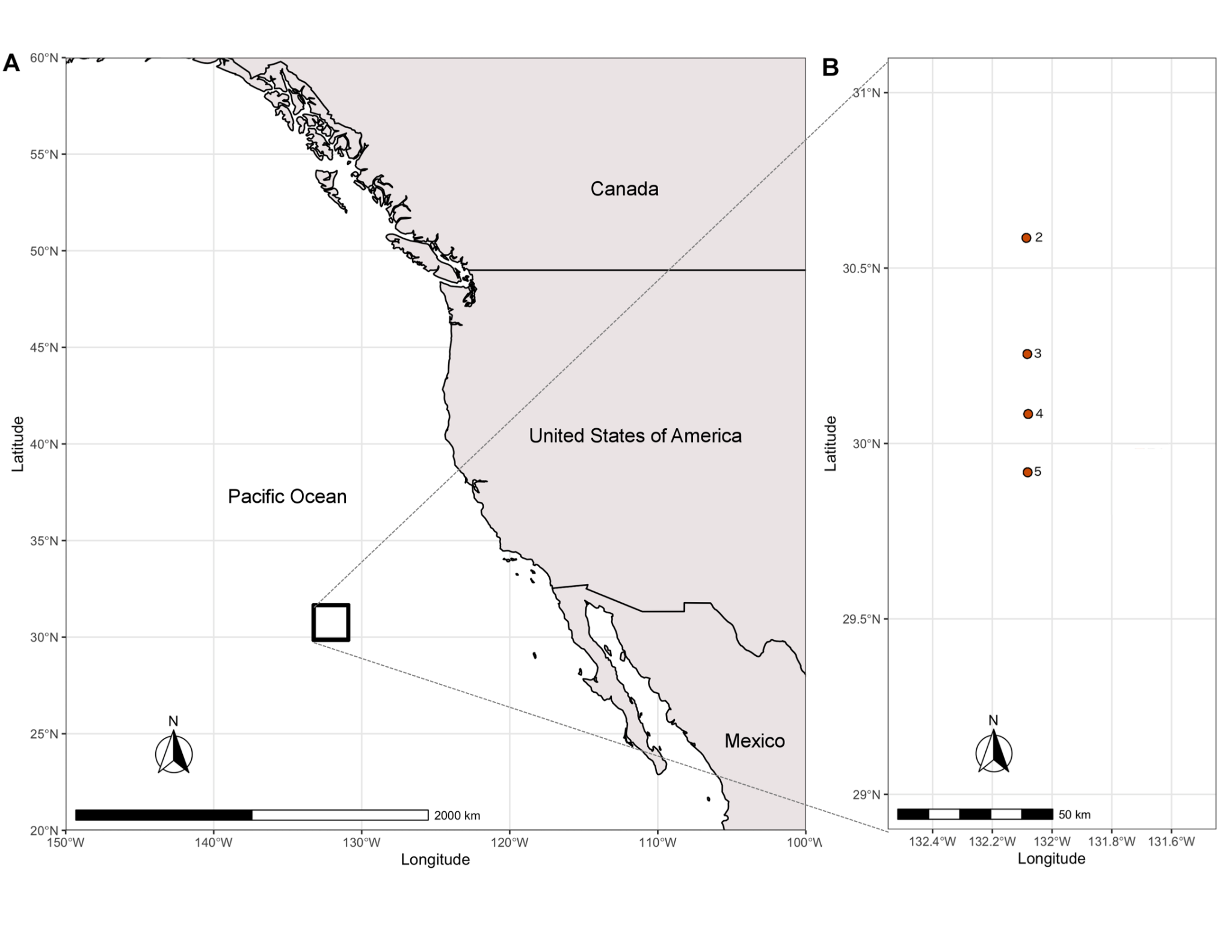


Supplementary Figure 1. Map of the sampling area and sampling sites, modified from previously published (Semedo *et al*., 2021) . (A) Map of North America and Pacific Ocean with sampling area denoted by a black rectangle. (B) Sampling sites (2, 3, 4, and 5) are identified by cast number. Information on casts and corresponding sample depths are shown in Table 1).


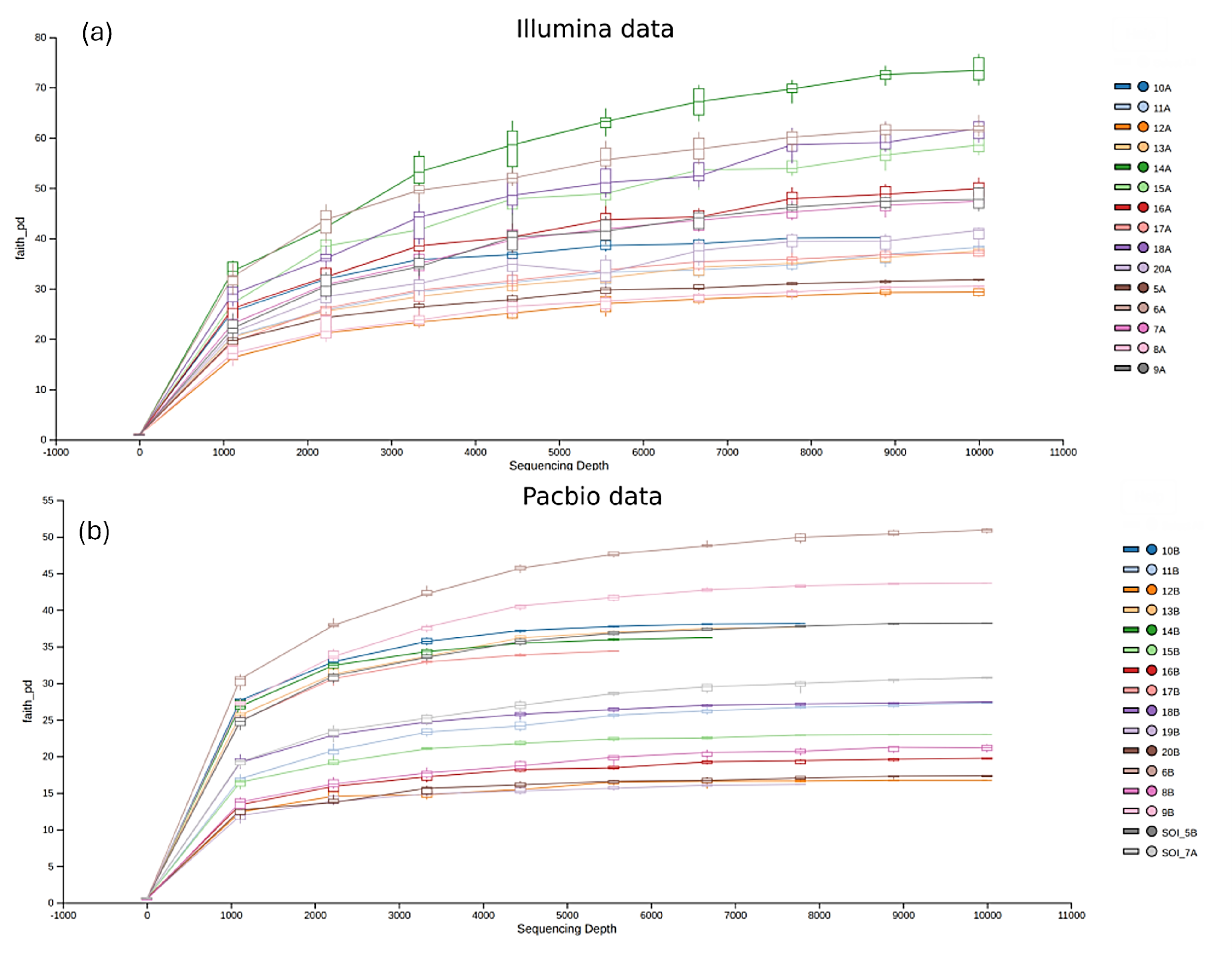


Supplementary Figure 2. Rarefaction curves of Illumina (a) and PacBio (b) sequencing methodologies, considering all sampling sites.

**Supplementary Tables**

| Supplementary Table 1. Complete DADA2 statistics of the Illumina sequencing. | | | | | | | | |
| --- | --- | --- | --- | --- | --- | --- | --- | --- |
| sample-id | input | filtered | % of input passed filter | denoised | merged | % of input merged | non-chimeric | %of input non-chimeric |
| #q2:types | numeric | numeric | numeric | numeric | numeric | numeric | numeric | numeric |
| S4_180m | 40801 | 18406 | 45.11 | 16305 | 10318 | 25.29 | 9801 | 24.02 |
| S4_108m | 56201 | 27997 | 49.82 | 26098 | 21387 | 38.05 | 19772 | 35.18 |
| S4_5m | 42451 | 20087 | 47.32 | 18692 | 15174 | 35.74 | 14158 | 33.35 |
| S3_500m | 91066 | 41100 | 45.13 | 39542 | 27558 | 30.26 | 26674 | 29.29 |
| S3_175m | 78690 | 34135 | 43.38 | 32275 | 22598 | 28.72 | 21183 | 26.92 |
| S3_122m | 72153 | 35600 | 49.34 | 32456 | 25375 | 35.17 | 23702 | 32.85 |
| S3_5m | 69639 | 33946 | 48.75 | 31760 | 26194 | 37.61 | 24554 | 35.26 |
| S2_500m | 51765 | 21247 | 41.05 | 19803 | 13175 | 25.45 | 12768 | 24.67 |
| S2_130m | 86160 | 41670 | 48.36 | 38401 | 29361 | 34.08 | 27144 | 31.5 |
| S2_5m | 56439 | 27197 | 48.19 | 25429 | 20221 | 35.83 | 19016 | 33.69 |
| S5_500m | 55018 | 26093 | 47.43 | 24662 | 17157 | 31.18 | 16546 | 30.07 |
| S5_200m | 63111 | 35494 | 56.24 | 32093 | 22837 | 36.19 | 21787 | 34.52 |
| S5_110m | 47128 | 23814 | 50.53 | 21716 | 16523 | 35.06 | 15364 | 32.6 |
| S5_5m | 38147 | 18720 | 49.07 | 17438 | 14779 | 38.74 | 13874 | 36.37 |
| S4_500m | 73651 | 31532 | 42.81 | 30400 | 22255 | 30.22 | 21393 | 29.05 |

| Supplementary Table 2. Complete DADA2 statistics on PacBio sequencing. | | | | | | | | |
| --- | --- | --- | --- | --- | --- | --- | --- | --- |
| sample-id | input | primer-removed | % of input primer-removed | filtered | % of input passed filter | denoised | non-chimeric | % of input non-chimeric |
| #q2:types | numeric | numeric | numeric | numeric | numeric | numeric | numeric | numeric |
| S4_180m | 20958 | 20899 | 99.72 | 15615 | 74.51 | 8050 | 8050 | 38.41 |
| S4_108m | 21143 | 21083 | 99.72 | 17054 | 80.66 | 13065 | 12972 | 61.35 |
| S4_5m | 21216 | 21122 | 99.56 | 16417 | 77.38 | 11190 | 11038 | 52.03 |
| S3_500m | 14498 | 14447 | 99.65 | 11575 | 79.84 | 8145 | 8092 | 55.81 |
| S3_175m | 21672 | 21571 | 99.53 | 15892 | 73.33 | 7302 | 7296 | 33.67 |
| S3_122m | 23739 | 23649 | 99.62 | 17983 | 75.75 | 10835 | 10771 | 45.37 |
| S3_5m | 20423 | 20363 | 99.71 | 16578 | 81.17 | 13028 | 13007 | 63.69 |
| S2_500m | 10151 | 10125 | 99.74 | 8257 | 81.34 | 5969 | 5933 | 58.45 |
| S2_130m | 22330 | 22247 | 99.63 | 17192 | 76.99 | 11463 | 11438 | 51.22 |
| S2_5m | 28844 | 28693 | 99.48 | 21810 | 75.61 | 14297 | 14148 | 49.05 |
| S5_200m | 26622 | 26542 | 99.7 | 21077 | 79.17 | 14484 | 14478 | 54.38 |
| S5_5m | 27413 | 27313 | 99.64 | 21443 | 78.22 | 15460 | 15255 | 55.65 |
| S4_500m | 19768 | 19723 | 99.77 | 15697 | 79.41 | 10152 | 10152 | 51.36 |
| S5_500m | 23662 | 23565 | 99.59 | 17891 | 75.61 | 10634 | 10590 | 44.76 |
| S5_110m | 26504 | 26408 | 99.64 | 20879 | 78.78 | 14910 | 14812 | 55.89 |

| Supplementary Table 3. Complete information on the sampling sites, including depth, coordinates and physicochemical parameters (Semedo *et al*., 2021) | | | | | | | | | | | | | |
| --- | --- | --- | --- | --- | --- | --- | --- | --- | --- | --- | --- | --- | --- |
| **Depth Layer** | **Sample ID** | **Cast** | **Depth (m)** | **Latitude** | **Longitude** | **Temp. (C)** | **Salinity (PSU)** | **Dissolved O_2_ (µM)** | **Turbiity (NTU)** | **Fluorescence (mg/m3)** | **NO_2_ (uM)** | **NO_3_ (uM)** | **NH_4_ (uM)** |
| **Surface (5 m)** | S5_5m | 2 | 5 | 29.918 | -132.082 | 19.1 | 34.7 | 162.78 | 0.057 | - 0.017 | 0.015 | 0.015 | 0.04 |
|  | S4_5m | 3 | 5 | 30.084 | -132.080 | 19.5 | 34.8 | 161.43 | 0.063 | - 0.017 | 0.015 | 0.015 | 0.04 |
|  | S3_5m | 4 | 5 | 30.255 | -132.083 | 19.5 | 34.8 | 161.38 | 0.057 | - 0.003 | 0.015 | 0.015 | 0.04 |
|  | S2_5m | 5 | 5 | 30.757 | -132.081 | 18.7 | 34.2 | 164.16 | 0.063 | 0.013 | 0.015 | 0.015 | 0.04 |
| **DCM  (108 - 130 m)** | S5_110m | 2 | 110 | 29.918 | -132.082 | 15.6 | 34.2 | 162.72 | 0.057 | 0.561 | 0.093 | 0.015 | 0.04 |
|  | S4_108m | 3 | 108 | 30.084 | -132.080 | 16.5 | 34.4 | 164.23 | 0.057 | 0.668 | 0.015 | 0.015 | 0.04 |
|  | S3_122m | 4 | 122 | 30.255 | -132.083 | 16.2 | 34.3 | 164.50 | 0.063 | 0.668 | 0.081 | 0.015 | 0.04 |
|  | S2_130m | 5 | 130 | 30.757 | -132.081 | 15.8 | 34.1 | 166.53 | 0.069 | 0.561 | 0.123 | 0.015 | 0.04 |
| **Below DCM (175 - 200 m)** | S5_200m | 2 | 200 | 29.918 | -132.082 | 10.6 | 33.8 | 138.76 | 0.045 | - 0.030 | 0.015 | 13.599 | 0.04 |
|  | S4_180m | 3 | 180 | 30.084 | -132.080 | 11.4 | 33.8 | 144.89 | 0.063 | 0.024 | 0.015 | 11.909 | 0.04 |
|  | S3_175m | 4 | 175 | 30.255 | -132.083 | 11.8 | 33.8 | 149.31 | 0.045 | 0.064 | 0.038 | 8.373 | 0.04 |
| **Mesopelagic (500 m)** | S5_500m | 2 | 500 | 29.918 | -132.082 | 5.9 | 34.1 | 25.98 | 0.045 | - 0.003 | 0.155 | 38.170 | 0.04 |
|  | S4_500m | 3 | 500 | 30.084 | -132.080 | 5.7 | 34.1 | 29.77 | 0.039 | 0.010 | 0.015 | 32.518 | 0.11 |
|  | S3_500m | 4 | 500 | 30.255 | -132.083 | 5.7 | 34.1 | 23.78 | 0.045 | - 0.003 | 0.015 | 38.770 | 0.04 |
|  | S2_500m | 5 | 500 | 30.757 | -132.081 | 5.8 | 34.1 | 27.66 | 0.032 | - 0.003 | 0.017 | 37.176 | 0.65 |

***In Silico* Primer Validation For The Study Of *Planctomycetota***

Different primers (Supplementary Table 4) were tested for their ability to capture *Planctomycetota* diversity in environmental samples. This test was divided in two phases, (1) used *SILVA PrimeTest* software (<https://www.arb-silva.de/search/testprime/>) offered by SILVA database, (2) used a reference dataset of *Planctomycetota* genomes. Both methods served to assess the ability of different primers to match the 16S RNA gene sequence of *Planctomycetota*.

Supplementary Table 4. Primers compared in the validation

| Primer Set | Primer Name | Primer Sequence | Reference |
| --- | --- | --- | --- |
| 1 | Pla46f | GGATTAGGCATGCAAGTC | Bengtsson & Øvreås, 2010 |
| 1 | 1542r | AAGGAGGTGATCCAGCCGCA | Bengtsson & Øvreås, 2010 |
| 2 | 58f | GGCATGGATTAGGCATGC | Kirkpatrick J et al., 2006 |
| 2 | 926r | CCACCGCTTGTGTGAGCCCC | Kirkpatrick J et al., 2006 |
| 3 | PLA352F | GGCTGCAGTCGAGRATCT | Mühling et al., 2008 |
| 3 | PLA920R | TGTGTGAGCCCCCGTCAA | Mühling et al., 2008 |
| 4 | 27F | AGRGTTYGATYMTGGCTCAG | Lane, 1991 |
| 4 | 1492R | RGYTACCTTGTTACGACTT | Paliy et al., 2009 |
| 5 | 515F | GTGYCAGCMGCCGCGGTAA | Apprill et al., 2015 |
| 5 | Y926R-jed | CCGYCAATTYMTTTRAGTTT | Parada et al., 2016 |

TestPrime allows the evaluation of the performance of primer pairs by running an *in silico* PCR on the SILVA databases. From the results of the PCR, TestPrime computes coverages for each taxonomic group in all of the taxonomies offered by SILVA. The analysis resulted in clear evidence that the pair 27F/1492R and 515F/Y926R-jed are the primers pair matching more *Planctomycetota* (Supplementary Table 5). Namely, the primers set with the better performance were 515F_Y926R-jed (85% of *Planctomycetota* retrieved), and 27F_1492R (69% of *Planctomycetota* retrieved).

Supplementary Table 5. SILVA TestPrime results

| primers | number of genomes matched | percentage of *Planctomycetota* |
| --- | --- | --- |
| 515F_Y926R-jed | 383508 | 85,00% |
| 27F_1492R | 70553 | 69,00% |
| PLA352F_PLA920R | 4836 | 55,85% |
| 58f_926r | 364 | 5,65% |
| pla46f_1542r | 0 | 0,00% |

A reference dataset of genomes composed of 130 *Planctomycetota* *genomes* was used to test a total of 5 pairs of primers (Supplementary Table 4). The number of matches of such primers were counted for reference genome, together with the total number of matches in the dataset per pair primers, resulting in Supplementary Table 6.

Supplementary Table 6. *In silico* PCR of reference genome dataset

| primers | number of genomes matched | total number of genomes | percentage |
| --- | --- | --- | --- |
| 27F_1492R | 77 | 129 | 59,69% |
| 515F_Y926R-jed | 74 | 129 | 57,36% |
| PLA352F_PLA920R | 67 | 129 | 51,94% |
| pla46f_1542r | 53 | 129 | 41,08% |
| 58f_926r | 30 | 129 | 23,26% |

The *in silico* PCR on curated reference *Planctomycetota* genomes supported the previous results from SILVA PrimeTest in 27F/1492R and 515F/Y926R-jed being the most suitable primers for the study of *Planctomycetota*.

**Supplementary bibliography**

Bengtsson M.M., Øvreås L. (2010) Planctomycetes dominate biofilms on surfaces of the kelp Laminaria hyperborea. BMC Microbiol 10:261 doi: 10.1186/1471-2180-10-261

Kirkpatrick, J. *et al.* Diversity and distribution of Planctomycetes and related bacteria in the suboxic zone of the Black Sea. *Applied and Environmental Microbiology* 72, 3079–3083 (2006).

Klindworth, A. *et al.* Evaluation of general 16S ribosomal RNA gene PCR primers for classical and next-generation sequencing-based diversity studies. *Nucleic Acids Research* 41, e1 (2013).

Lane D.J. (1991) 16S/23S rRNA sequencing. In: & SE, M. G (eds) Nucleic Acid Techniques in Bacterial Systematics pp 115-175

Menzel, P., Ng, K. L. & Krogh, A. Fast and sensitive taxonomic classification for metagenomics with Kaiju. *Nature Communications* 7: 11257 (2016).

Mühling, M., Woolven-Allen, J., Murrell, J. C. & Joint, I. Improved group-specific PCR primers for denaturing gradient gel electrophoresis analysis of the genetic diversity of complex microbial communities. *ISME Journal* 2, 379–392 (2008).
